# Editorial: Contribution of phenylpropanoid metabolism to plant development and stress responses

**DOI:** 10.3389/fpls.2024.1456913

**Published:** 2024-08-14

**Authors:** Chaofeng Li, Yuanzhong Jiang, Changzheng Xu, Xiupeng Mei

**Affiliations:** ^1^ Maize Research Institute, Southwest University, Chongqing, China; ^2^ Engineering Research Center of South Upland Agriculture, Ministry of Education, Southwest University, Chongqing, China; ^3^ Key Laboratory for Bio-resources and Eco-environment of Ministry of Education, College of Life Science, Sichuan University, Chengdu, China; ^4^ Chongqing Key Laboratory of Tree Germplasm Innovation and Utilization, Integrative Science Center of Germplasm Creation, School of Life Sciences, Southwest University, Chongqing, China

**Keywords:** phenylpropanoid metabolism, lignin, flavonoids, multiomics, icariin

Phenylpropanoids are specialized secondary metabolites from land plants, which contribute to plant development and stress responses. As part of the shikimate pathway, phenylpropanoid metabolism produce nearly 10,000 metabolites, such as lignin, flavonoids, chlorogenic acids, phenolic glycosides, etc (Dong and Lin, 2021). Meanwhile, flavonoids contribute to organ pigmentation, plant signal communication and reproduction, UV protection, plant-microbe interactions, and plant protection against pathogens and herbivores, while lignin have been demonstrated to be involved in mechanical support and waterproofing of plants (Deng and Lu, 2017). Additionally, phenylpropanoids also have numerous pharmacological and industrial applications, such as producing perfumes, pharmaceuticals, and biopolymers (Lin and Eudes, 2020), and potential targets for carbon sequestration in soil and biomass (Eckardt et al., 2023). This Research Topic encompasses six original research articles and one review article focusing on phenylpropanoid metabolism to plant development and stress responses.

Over the past decades, molecular regulatory mechanisms of lignin biosynthesis have been extensively studied, and a NAC/MYB mediated hierarchical regulatory network has been established (Li et al., 2024). Zhao et al. found that jasmonic acid (JA) could inhibit lignin deposition and identified a JA signal inhibitor PtoJAZ5, which participates in the regulation of secondary vascular development of *Populus*. Overexpression of PtoJAZ5 in poplar and Arabidopsis inhibited secondary cell wall (SCW) thickening and down-regulated the expression of SCW biosynthesis-related genes. PtoJAZ5 has interactions with NAC/MYB master regulators, such as MYB2/3/74, and WND3A/4A/6A/6B, and their interactions inhibit the expression of

SCW related genes. Tang et al. isolated and overexpressed poplar *PtoMYB031* in wild-type *Populus tomentosa.* And they found that overexpression of MYB31 significantly reduced lignin content, and inhibited vascular development in stems, resulting in decreased SCW thickness in xylem tissues. Hence, PtoMYB031 is found to have interaction with PtoZAT11 and forms a complex to suppress the expression of some key NAC regulators, PtoWND1A and PtoWND2B. This work was further validated by a recent study of the same MYB transcription factor in *Populus alba* × *Populus glandulosa* (Zhang et al., 2024). These findings enhance our understanding of the transcriptional regulation of SCW formation, especially the lignin accumulation, and greatly enrich the hierarchical regulatory network.

A range of intense extreme weather events, including heat, drought, salinity, are now increasing far outside the historical climate. Secretion of phenylpropanoid compounds is an effective strategy for plants to adapt to changes in their environment. In Cassava, Wang et al. indicated that plants could enhance lignin biosynthesis and improve pyruvate synthesis to response to high temperature. *In situ* expression of the key rate-limiting enzyme MeCCoAOMT within mid-vein xylem vessels further demonstrated the importance of lignin pathway under high temperature stress. In grape, Zhao et al. revealed that high salinity inhibited cell growth and enhanced the accumulation of proanthocyanidins (PAs) and anthocyanins more than low salinity. The research also indicated that the anthocyanin pathway was more sensitive to salt concentration than the PA pathway, and revealed their coordination between flavonoid biosynthesis and cell wall metabolism to response to salinity stress. Meanwhile, Ghitti et al. explored the roles of flavonoids in root community recruitment and assembly involving non-symbiotic beneficial interactions under polychlorinated biphenyls (PCB) stress. And they found that flavonoids play a prominent role during the early events of bacterial colonization of *Paraburkholderia xenovorans* LB400 under normal or PCB stress. These studies provide new insights into the roles of phenylpropanoid compounds under biotic and abiotic stresses.

Multi-Omics studies bring more tools and methods for the studies of plant growth and development, and also supply new possibility for exploring the special compounds. Zhu et al. integrated the metabolomic, transcriptomic, and proteomic to explore the regulatory network of a special chemical compound of flavonoids, icariin, in *Herba Epimedii*. They found 32 and 66 of the candidate genes belonged to the anthocyanin and flavonoid pathways, of which 5 genes are icariin-specific regulators. And the results prepare valuable gene resources for further research and development in phenylpropanoid compounds and medicinal plant cultivation. Gao et al. reviewed the application and development of mathematical models in plant Multi-Omics studies. The paper introduces the relevant principles and types of bioinformatics tools for plant metabolomics to summarize how mathematical models can be used to solve specific problems in plant secondary metabolism. Additionally, the review also highlights the significance of interdisciplinary collaboration using mathematical models, which may improve our understanding of phenylpropanoid metabolism in plants.

The studies in the current Research Topic have greatly advanced our understanding of phenylpropanoid metabolic regulation to plant development and stress responses, especially the lignin and flavonoids. In the future, the ability to bridge the fields of molecular biology, biochemistry, epigenetics, bioinformatics, and multi-omics studies will be crucial in resolving the complexity of metabolic regulatory networks of phenylpropanoids.
